# Adaptive immune response to BNT162b2 mRNA vaccine in immunocompromised adolescent patients

**DOI:** 10.3389/fimmu.2023.1131965

**Published:** 2023-03-27

**Authors:** Guy Bader, Michal Itan, Liat Edry-Botzer, Hadar Cohen, Orly Haskin, Yael Mozer-Glassberg, Liora Harel, Ariel Munitz, Nufar Marcus Mandelblit, Motti Gerlic

**Affiliations:** ^1^ Department of Clinical Microbiology and Immunology, Sackler School of Medicine Tel Aviv University, Tel Aviv, Israel; ^2^ Sackler School, Medicine Tel Aviv University, Tel Aviv, Israel; ^3^ Nephrology Unit, Schneider Children’s Medical Center of Israel, Petach Tikva, Israel; ^4^ Gastroenterology Unit, Schneider Children’s Medical Center of Israel, Petach Tikva, Israel; ^5^ Rheumatology Unit, Schneider Children’s Medical Center of Israel, Petach Tikva, Israel; ^6^ Kipper Institute of Immunology, Schneider Children’s Medical Center of Israel, Petach Tikva, Israel

**Keywords:** transplantation, immunodeficiency - primary, vaccination, COVID-19, adaptive immunity

## Abstract

Protective immunity against COVID-19 is orchestrated by an intricate network of innate and adaptive anti-viral immune responses. Several vaccines have been rapidly developed to combat the destructive effects of COVID-19, which initiate an immunological cascade that results in the generation of neutralizing antibodies and effector T cells towards the SARS-CoV-2 spike protein. Developing optimal vaccine-induced anti-SARS- CoV-2 protective immunity depends on a fully competent immune response. Some evidence was gathered on the effects of vaccination outcomes in immunocompromised adult individuals. Nonetheless, protective immunity elicited by the Pfizer Biontech BNT162b2 vaccine in immunocompromised adolescents received less attention and was mainly focused on the antibody response and their neutralization potential. The overall immune response, including T-cell activities, was largely understudied. In this study, we characterized the immune response of vaccinated immunocompromised adolescents. We found that immunocompromised adolescents, which may fail to elicit a humoral response and develop antibodies, may still develop cellular T-cell immunity towards SARS-CoV-2 infections. Furthermore, most immunocompromised adolescents due to genetic disorders or drugs (Kidney and liver transplantation) still develop either humoral, cellular or both arms of immunity towards SARS-CoV-2 infections. We also demonstrate that most patients could mount a cellular or humoral response even after six months post 2^nd^ vaccination. The findings that adolescents immunocompromised patients respond to some extent to vaccination are promising. Finally, they question the necessity for additional vaccination boosting regimens for this population who are not at high risk for severe disease, without further testing of their post-vaccination immune status.

## Introduction

The severe acute respiratory syndrome coronavirus 2 (SARS-CoV-2) was identified in late 2019 and described as causing a pneumonia outbreak, known as coronavirus-induced disease-19 (COVID-19) (1). COVID-19 has affected and still affects millions of people worldwide, resulting in mortality and morbidity rates as well as high healthcare costs, difficulties in treatment (2), and overwhelming economic burden that resulted in the loss of numerous additional lives and extensive long-term damage (3).

Protective immunity against COVID-19 is orchestrated by an intricate network of innate and adaptive anti-viral immune responses (4). Initially, SARS-CoV-2 infection triggers a local inflammatory response that recruits neutrophils and monocytes to the lungs and is accompanied by the release of multiple cytokines including IL-1β, IL-6, TNF-α, IL-12 and interferons (α, β and γ) (5, 6). Subsequently, activation of antigen presentation processes primes an adaptive T and B cell response that generates neutralizing antibodies and effector T cells that can recognize and kill virally infected cells (4). In most cases this process can resolve the infection. However, in some cases, a dysfunctional immune response can cause severe lung and even systemic pathology (7). If a protective inflammatory response does not occur, a cytokine storm could develop, resulting in multiple organ failure (8). Patients with various co-morbidities aged over 60 years are more likely to develop such a dysfunctional immune response that causes pathology and fails to successfully eradicate the pathogen (9).

To combat the destructive effects of COVID-19, several vaccines have been rapidly developed, including the Pfizer-Biontech BNT162b2 mRNA vaccine (10, 11). In this vaccine, mRNA encoding the SARS-CoV-2 spike (S) protein was encapsulated in lipid nanoparticle vectors encoding the viral S protein (12). Injection of the encapsulated mRNA results in the production of high levels of S protein. Following vaccination, S protein-specific memory T cells and B cells develop and circulate along with high-affinity SARS-CoV-2 antibodies, which jointly act to prevent SARS-CoV-2 infection and disease (13). Thus, developing optimal vaccine-induced anti-SARS-CoV-2 protective immunity depends on a fully competent immune response.

The decision to vaccinate immunocompromised patients is not trivial, especially in adolescent patients, which are not at high risk of developing severe disease (14, 15). On the one hand, recent reports (primarily in adult patients) have suggested that immunocompromised patients might display an increased risk of developing severe COVID-19 (16, 17). Thus, these patients should be prioritized for vaccination. In contrast, their underlying immune deficiency or immunosuppressive treatment might impair their ability to respond to the vaccine and develop protective immunity, characterized by generating anti-SARS-CoV-2 antibodies and cellular immune responses (5, 13). Some evidence exists regarding the effects of vaccination outcomes in adult immunocompromised individuals (18), while the protective immunity elicited by vaccines in immunocompromised children was mainly focused on the humoral response (19, 20). In one study, it was shown that adolescents who are immunocompromised (post-transplantation, cancer, or due to immunosuppressive drugs) displayed impaired *in vitro* neutralization capacity as measured by competition with ACE2, and total IgG against RBD, in comparison to adolescents who have chronic diseases, such as HIV. Another study, which focused on adolescents with inflammatory bowel disease (IBD), found no difference in antibody neutralization capacity as measured by competition with ACE2 (neutralization *in-vitro*) between IBD patients and healthy adolescents. However, combination therapy (anti-tumor necrosis factor-α + immunomodulators) showed significantly reduced neutralization capacity relative to those in other therapies and controls. Nevertheless, and most importantly, the breakthrough infection was similar between all groups without statistical differences. These results emphasize the need to characterize not only the humoral immune response, but also the cellular immune response of adolescent patients following SARS-CoV-2 vaccination since this will enhance our understanding of the potential degree of protection that these patients have. Furthermore, it will enable the design of an optimal immunization regimen for this patient population. This is especially important for future vaccination regimens utilizing relevant sequences of viral S proteins.

## Results

**Figure 1:**
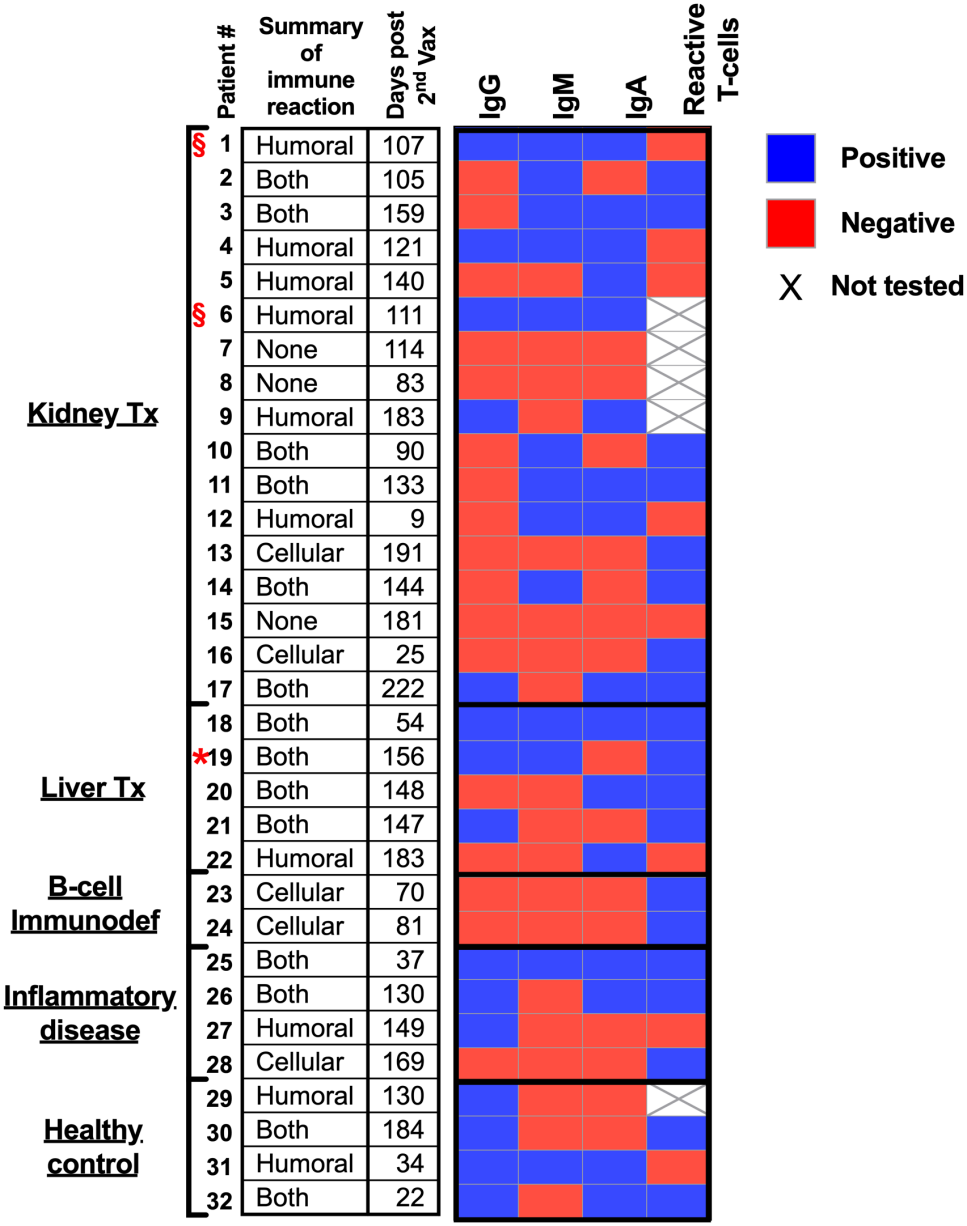
Humoral and cellular responses to BNT162b2 mRNA vaccine in immunocompromised adolescent patients.

Herein, we hypothesize that immunocompromised adolescent patients, which fail to elicit a humoral response and develop antibodies, will still develop cellular T-cell immunity toward SARS-CoV-2 infections. A cohort of 17 kidney transplant patients (two of which were not immunosuppressed at the time of vaccination), five liver transplant patients, two B-cell deficient patients, and four inflammatory disease patients that received two doses of the BNT162b2 mRNA vaccine were recruited (**Table 1**). Their humoral immune responses were determined using a flow cytometry ELISA capable of recognizing anti-spike IgG, IgM, and IgA simultaneously (**Supplemental Figure 1**), similar to our recent study on Covid-19 patients (6). Their cellular immune response was determined by an IFN-γ ELISPOT of isolated mononuclear cells stimulated with N- and S SARS-CoV-2 peptides. Even though the patients displaying B-cell deficiencies were unable to generate antibodies in response to BNT162b2 mRNA vaccination, they all generated a cellular response (**Figure 1** and **Supplemental Figure 2A** and **Supplemental Figure 3**). Thus, they may still benefit from vaccination since it triggers the cellular arm, which is most important in eliciting protective immunity against SARS-CoV-2 infections (21). Assessment of humoral and cellular immunity in a cohort of immunocompromised adolescent patients, which were under a medication regimen that generally suppressed innate immune responses or suppressed their T cell or B cell responses (**Table 1**), revealed that vaccination with BNT162b2 could induce either a humoral or cellular immune response. It should be noted that out of the 26 immunocompromised adolescent patients (two with B cell deficiency diseases), 18 subjects generated a partial antibody response, from whom only 9 subjects generated IgG. On the other hand, out of the 23 immunocompromised adolescent patients that were tested for T cell response, 17 subjects generated a positive response. In fact, out of the 23 immunocompromised adolescent patients, which we assessed for both B and T cell mediated response, only one did not develop a humoral or cellular immune response. This patient (patient number 15) was already six months after his 2^nd^ vaccination dose. Thus, his antibody levels may have naturally declined, as we have shown for patients in the past (6) and as suggested by plotting the patient data on a timeline (**Supplemental Figure 2B**). It should be noted that the decline that is shown by the semilog scale in **Supplemental Figure 2B** is not statistically significant. This is likely due to the fact that the majority of samples were taken 12 weeks or more after the 2^nd^ vaccination, where antibody response was already shown to decline by others (22, 23). Nevertheless, these patients may still have a memory response (22, 23).

**Table 1:** immunocompromised adolescent patients recruited in this study.

P#	Clinical dignosis	Age at 2nd vaccination	Date of 2nd vaccination	Date of blood collection	Time from 2nd dose in days	Medications (mode of action)										
						Suppresses innate immune responses			B-cell inhibition	T-cell inhibition	B & T-cell inhibition		Imuunoglobulin	Anti bacterial	Anti viral	mTOR blocker
						Steroids	anti TNFα	Prednisolone	Rituximab	Tacrolimus	Mycophenolate	Methotrexate	IVIG	Azithromycin	Zidovudine	Everolimus
1	Received the vaccination while being in a kidney failure before transplantation. Immunosuppressive treatment started five weeks post 2nd vaccination	16.9	05/03/2021	20/06/2021	107.00	+				+	+					
2	Kidney ANCA vasculitis - After kidney transplantation	17	07/03/2021	20/06/2021	105.00			+	+	+	+					
3	After kidney transplantation	19.5	26/01/2021	04/07/2021	159.00	+				+	+					
4	After kidney transplantation	16.9	05/03/2021	04/07/2021	121.00	+				+	+					
5	Joubert syndrome - After 2nd kidney transplantation	18.5	21/02/2021	11/07/2021	140.00	+				+	+					
6	Chronic kidney disease (CKD) - After kidney transplantation	15	05/04/2021	25/07/2021	111.00											
7	Lymphoma - After kidney transplantation	18.5	02/04/2021	25/07/2021	114.00	+				+	+					
8	Gorlin syndrome - After kidney transplantation	15.5	03/05/2021	25/07/2021	83.00	+				+						+
9	Cerebral palsy (CP) - After kidney transplantation	16.5	23/01/2021	25/07/2021	183.00	+				+	+					
10	Gorlin syndrome - After kidney transplantation	15.5	03/05/2021	01/08/2021	90.00	+				+						+
11	Posterior urethral valves (PUV) - After kidney transplantation	13	21/03/2021	01/08/2021	133.00	+				+	+					
12	Genetic nephrotic syndrome - After kidney transplantation	12	23/07/2021	01/08/2021	9.00	+				+	+					
13	Genetic nephrotic syndrome - After kidney transplantation	20	22/01/2021	01/08/2021	191.00	+				+	+					
14	Posterior urethral valves (PUV) - After kidney transplantation	16.3	25/03/2021	16/08/2021	144.00	+				+	+					
15	Genetic nephrotic syndrome - After kidney transplantation	20	16/02/2021	16/08/2021	181.00	+				+	+					
16	After kidney transplantation	13	15/01/2021	09/02/2021	25	+				+	+					
17	After kidney transplantation	14	02/07/2020	09/02/2021	222	+				+	+					
																
18	After liver transplantation	15.11	27/04/2021	20/06/2021	54.00					+						
19	After liver transplantation	17.7	לא חוסן - חלה	20/06/2021	156.00					+						
20	After liver transplantation	18.1	31/01/2021	28/06/2021	148.00					+	+					
21	After liver transplantation	17.5	07/02/2021	04/07/2021	147.00					+						
22	After liver transplantation	19		09/08/2021	183.00					+	+					
																
23	CVID disease	14	11/04/2021	20/06/2021	70.00				+				+		+	
24	X-Linked Agammaglobulinemia disease	16	07/04/2021	27/06/2021	81.00								+	+		
																
25	Immunodeficiency-WAS	12	03/07/2021	09/08/2021	37.00											
26	Bone marrow transplantation	14.2	08/04/2021	16/08/2021	130.00											
27	Crohn’s disease	13.4	20/03/2021	16/08/2021	149.00		+									
28	Juvenile idiopathic arthritis (JIA) associated uveitis	17	02/01/2021	20/06/2021	169.00		+					+				
																
29	Healthy control	12.5	08/04/2021	16/08/2021	130.00											
30	Healthy control	16.8	13/02/2021	16/08/2021	184.00											
31	Healthy control	13.11	13/07/2021	16/08/2021	34.00											
32	Healthy control	13.11	25/07/2021	16/08/2021	22.00											

## Discussion

Our study bears several limitations. Our relatively low sample size limits the ability of appropriate statistical analysis, especially when stratifying each subgroup of patients according to their medication, immunopathology, and affected organs. Second, our patient’s recruitment post-vaccination was done randomly, the time post their 2^nd^ vaccination may be too long, which may lead to a false negative conclusion, especially regarding their humoral immune response. Furthermore, as we have shown for patients who developed a mild disease following SARS-CoV-2 infection (6), a general statistical cutoff for determining whether a patient developed antibodies following infection using ROC analysis can be misleading. This is due to the fact that each patient has a different antibody titer baseline, and therefore, patients with a mild response, who may develop low antibody titers, may still develop antibodies that are above their personal baseline levels but not above the positivity threshold that was set by ROC analysis. Similarly, immunocompromised patients may develop mild antibody titers that would not be considered positive by ROC analysis-based methods. We suggest that, when possible, antibody titer (and maybe even T cell responses) should be determined prior to vaccination to set the individual background for each immunocompromised patient. These limitations can also explain the diversity and unpredicted response in all the immunocompromised adolescents, except for the B-cell deficiency. Despite these limitations, our data demonstrate that most patients could mount cellular or humoral responses. Collectively, these data are promising and question the necessity for additional vaccination boosts for adolescents who are not at high risk for severe disease (14, 15). Further studies that will focus on *in-vivo* neutralization assays, functionality of T-cell responses (e.g., CD4 vs CD8 responses) as well as monitoring the risk of these patients to infection and/or severe disease are required. This will enable comprehensive understanding of the immune responses generated upon vaccination especially in these patient populations.

Peripheral blood from immunocompromised adolescent patients was collected and separated into PBMCs and plasma. SARS-Cov2 RBD IgG/IgA/IgM antibodies were analyzed using iQue^®^ SARS-CoV-2 (IgG, IgM and IgA) Kit (Sartorius). Reactive T-cells were analyzed using T-SPOT^®^ Discovery SARS-CoV-2 (Oxford Immunotec).

Red star – Patient 19 was positive for infection with covid19. § symbol – not immunocompromised at time of vaccination (see **Table 1**).

## Material and methods

### Patients and their sample collection

Peripheral blood was obtained (~ 5 ml) from each patient during a routine check-up in the clinic at Schneider children’s medical center of Israel. All experiments were reviewed and approved by the Ethics committee of the Schneider children’s medical center (IRB#0209-21-RMC) and were performed according to their regulations and guidelines. Written informed consent to participate in this study was provided by all subjects or by the participants’ legal guardian/next of kin.

### Plasma and PBMCs preparation

Whole blood was centrifuged in EDTA (500×g, 5 min) in secure buckets. The supernatant was transferred into a clean 1.7/2 ml Eppendorf tube. The samples (plasma) were apportioned into 50 μl aliquots and stored at − 20°C or − 80°C for later use in iQue^®^ SARS-CoV-2 (IgG, IgM and IgA) Kit. The pellet was resuspended in RPMI (Biological Industries, Beit-Haemek, Israel), and PBMCs were isolated by density-gradient centrifugation using Histopaque-1077 (Sigma-Aldrich, 10771), as previously reported (24).

### Anti SARS-CoV-2 serological testing

Quantifications of IgG, IgM and IgA against SARS-CoV-2 RBD antigen were measured in subjects’ plasma (diluted 1:100) according to the protocol of iQue^®^ SARS-CoV-2 (IgG, IgM, and IgA) Kit (Sartorius).

### Measurement of T-cell response to SARS-CoV-2 peptides

Isolated PBMCs from subjects were used for reactive T-cell assays according to the T-SPOT^®^ Discovery SARS-CoV-2 protocol (Oxford Immunotec).

### Data analysis

Data were calculated using GraphPad Prism 9, and details can be found in the figure legends.

## Data availability statement

The original contributions presented in the study are included in the article/**Supplementary Material**. Further inquiries can be directed to the corresponding authors.

## Ethics statement

All experiments were reviewed and approved by the Ethics committee of the Schneider children’s medical center (IRB#0209-21-RMC) and were performed according to their regulations and guidelines. Written informed consent to participate in this study was provided by all subjects or by the participants’ legal guardian/next of kin.

## Author contributions

GB, MI, L-EB, and HC, performed experiments; GB, MI, AM, NM, and MG designed the experiments; HO, YM-G, LH, and NM, drew blood and provided patient characteristics; GB, AM, NM, and MG analyzed the data; AM, and MG, wrote and edited the manuscript; AM, and MG, supervised the work. All authors contributed to the article and approved the submitted version.

## Supplementary material

Supplementary Figure 1 Determining the cutoff for positivity and negativity values using the iQue^®^ SARS-CoV-2 (IgG, IgM and IgA) Kit. **(A-C)** Peripheral blood was collected from hospitalized COVID-19 patients (at least 14 days post symptoms (DPS)) and anonymous recovered patients (IgG n=103, IgM n=56, IgA n=54). Negative samples were obtained from true SARS-CoV-2 negative patients (i.e., prior to the SARS-CoV-2 pandemic) (IgG n=128, IgM n=128, IgA n=128). Plasma was obtained, diluted 1:100, and prepared according to the protocol of the iQue^®^ SARS-CoV-2 Kit. Data were calculated using GraphPad Prism 9; the dotted line represents the calculated cutoff value discriminating between positive and negative samples (specificity and sensitivity are shown for each antibody). An unpaired t-test with Welch’s correction was performed. P values are shown. Data were calculated using GraphPad Prism 9; the dotted line represents the calculated cutoff value discriminating between positive and negative samples.

Supplementary Figure 2 Immunocompromised adolescents’ individual anti-SARS-CoV-2-RBD antibodies response. **(A)** Immunocompromised adolescents’ individual anti-SARS-CoV-2-RBD antibodies. Individual IgG (blue), IgM (red), and IgA (green) levels of each of the immunocompromised adolescents were plotted. **(B)** Individual IgG (blue), IgM (red), and IgA (green) levels of each of the immunocompromised adolescents were plotted according to the time post 2^nd^ vaccination. Data were calculated using GraphPad Prism 9; the dotted line represents the calculated cutoff value discriminating between positive and negative samples. Solid lines - antibody kinetics was evaluated by calculating the nonlinear regression (fitting method - least square regression). Line’s slopes and R squares were S=-0.005 and R^2 =^ 0.155 for IgG, S=-0.001 and R^2 =^ 0.016 for IgM, and -S=0.007 and R^2 =^ 0.39 for IgA.

Supplementary Figure 3 Immunocompromised adolescents’ individual T-cell response to SARS-CoV-2 peptides. Isolated PBMCs from Immunocompromised adolescents’ individual were used for reactive T-cell assays according to the T-SPOT^®^ Discovery SARS-CoV-2 protocol. Spots were counted for positive control, negative control, and either Covid-19 spike peptides or nucleocapsid and plotted using GraphPad Prism 9. Representative wells and spots of four different patients are shown.
