# Supplementary figures and images for: Adaptive immune response to BNT162b2 mRNA vaccine in immunocompromised adolescent patients
Source: Front Immunol. 2023 Mar 27;14:1131965. doi: 10.3389/fimmu.2023.1131965 (PMC10084476; doi:10.3389/fimmu.2023.1131965)

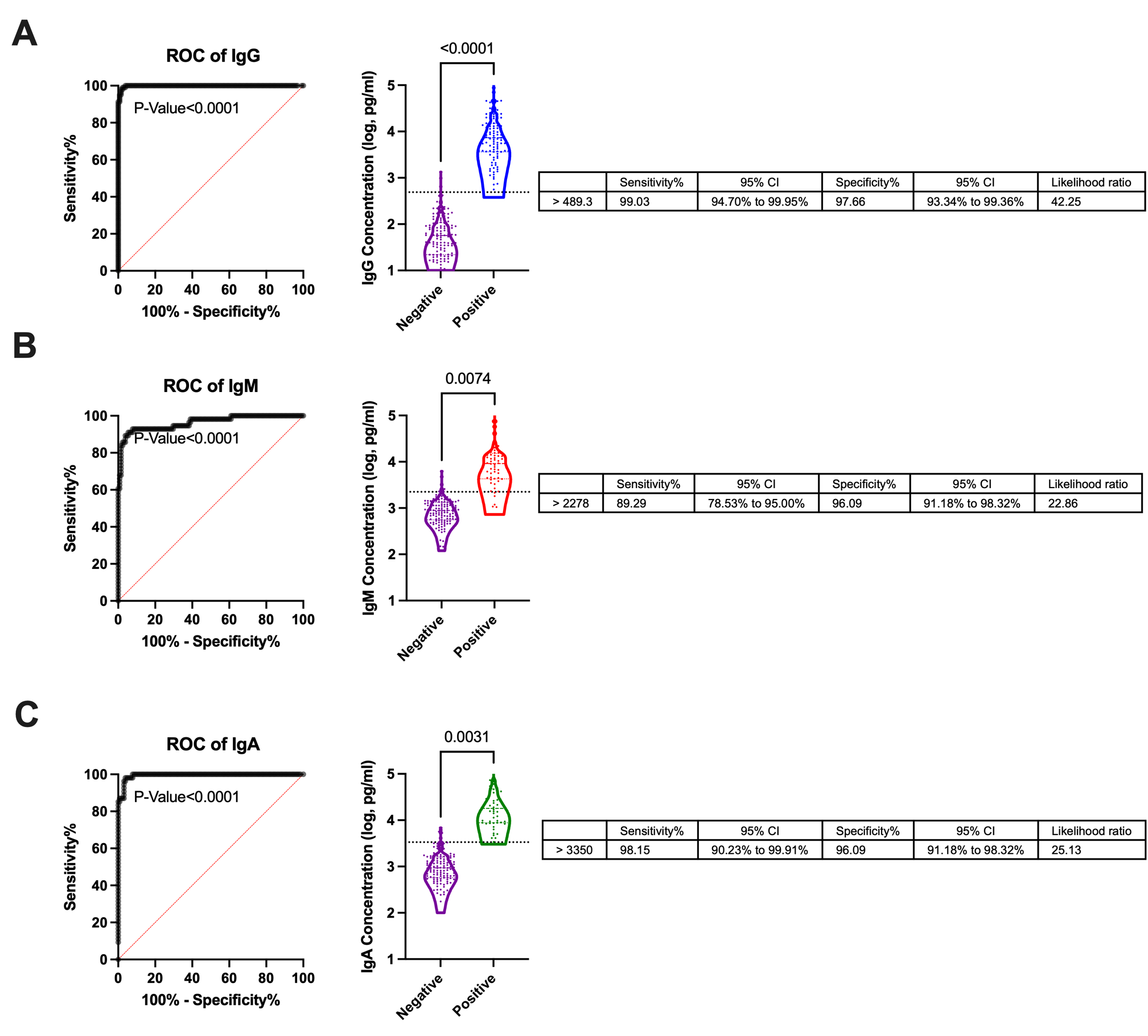

Supplement: Supplementary Figure 1 — Determining the cutoff for positivity and negativity values using the iQue® SARS-CoV-2 (IgG, IgM and IgA) Kit. (A-C) Peripheral blood was collected from hospitalized COVID-19 patients (at least 14 days post symptoms (DPS)) and anonymous recovered patients (IgG n=103, IgM n=56, IgA n=54). Negative samples were obtained from true SARS-CoV-2 negative patients (i.e., prior to the SARS-CoV-2 pandemic) (IgG n=128, IgM n=128, IgA n=128). Plasma was obtained, diluted 1:100, and prepared according to the protocol of the iQue® SARS-CoV-2 Kit. Data were calculated using GraphPad Prism 9; the dotted line represents the calculated cutoff value discriminating between positive and negative samples (specificity and sensitivity are shown for each antibody). An unpaired t-test with Welch’s correction was performed. P values are shown. Data were calculated using GraphPad Prism 9; the dotted line represents the calculated cutoff value discriminating between positive and negative samples. [file Image_1.tiff]

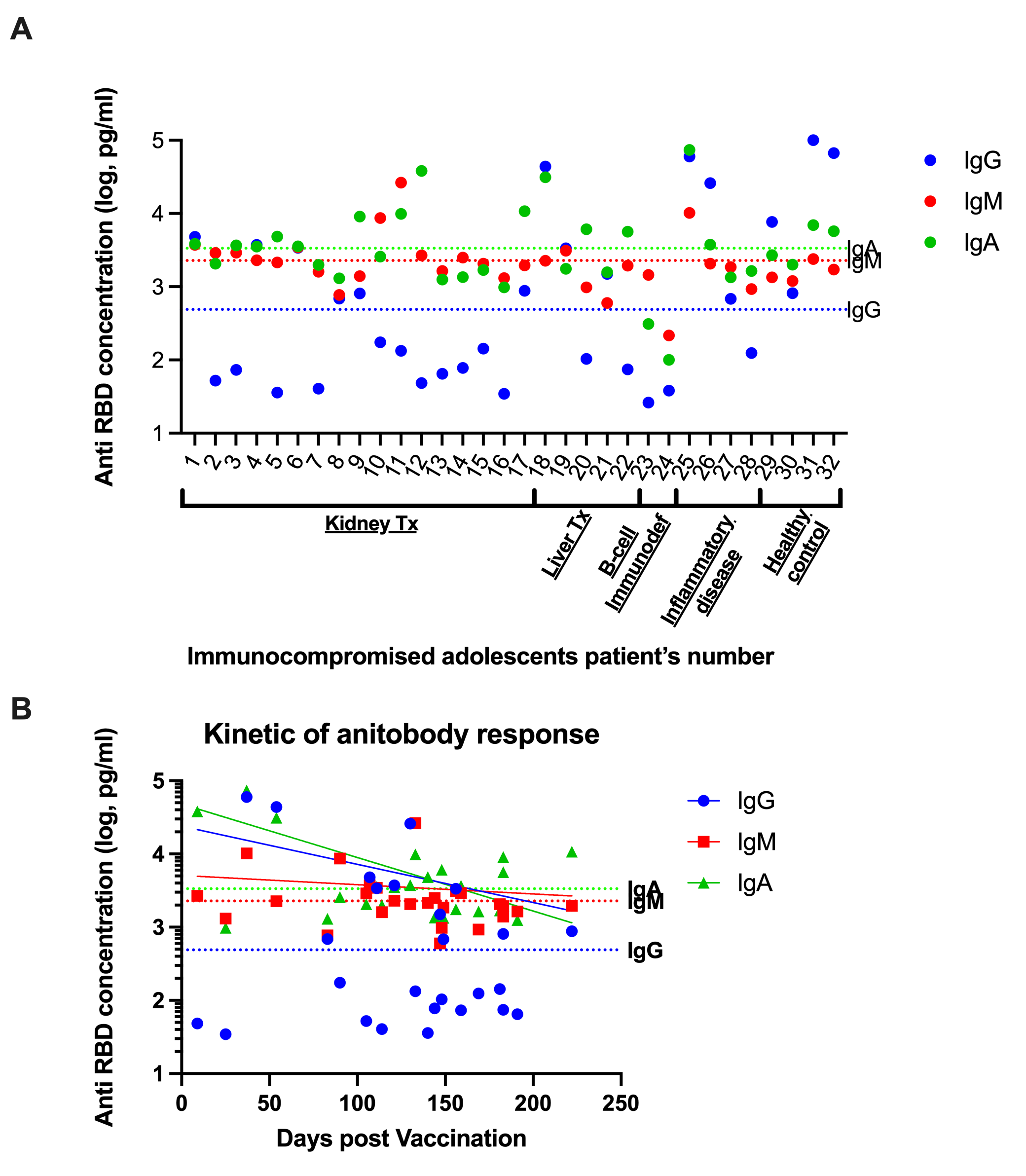

Supplement: Supplementary Figure 2 — Immunocompromised adolescents’ individual anti-SARS-CoV-2-RBD antibodies response. (A) Immunocompromised adolescents’ individual anti-SARS-CoV-2-RBD antibodies. Individual IgG (blue), IgM (red), and IgA (green) levels of each of the immunocompromised adolescents were plotted. (B) Individual IgG (blue), IgM (red), and IgA (green) levels of each of the immunocompromised adolescents were plotted according to the time post 2nd vaccination. Data were calculated using GraphPad Prism 9; the dotted line represents the calculated cutoff value discriminating between positive and negative samples. Solid lines - antibody kinetics was evaluated by calculating the nonlinear regression (fitting method - least square regression). Line’s slopes and R squares were S=-0.005 and R2 = 0.155 for IgG, S=-0.001 and R2 = 0.016 for IgM, and -S=0.007 and R2 = 0.39 for IgA. [file Image_2.tiff]

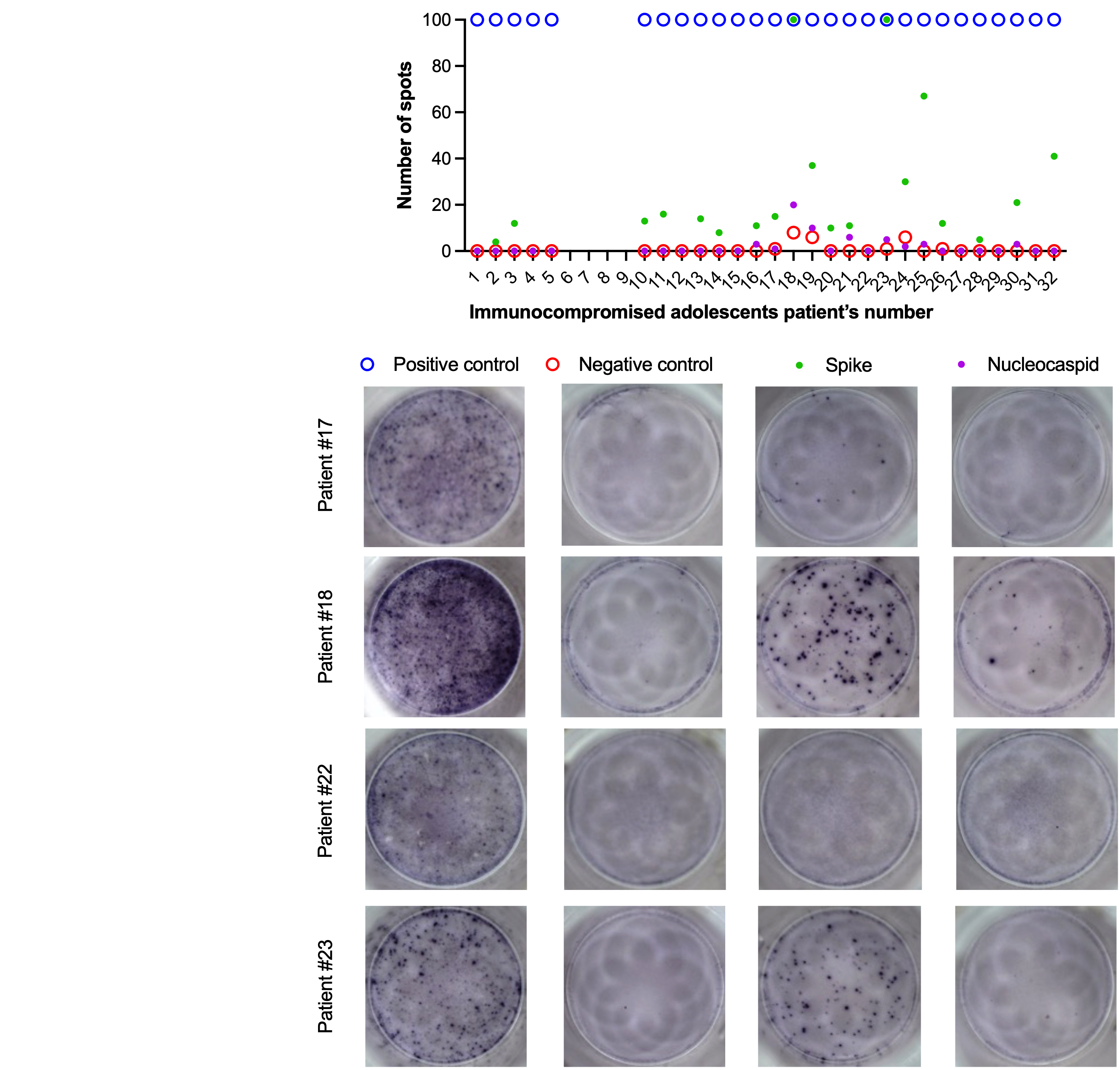

Supplement: Supplementary Figure 3 — Immunocompromised adolescents’ individual T-cell response to SARS-CoV-2 peptides. Isolated PBMCs from Immunocompromised adolescents’ individual were used for reactive T-cell assays according to the T-SPOT® Discovery SARS-CoV-2 protocol. Spots were counted for positive control, negative control, and either Covid-19 spike peptides or nucleocapsid and plotted using GraphPad Prism 9. Representative wells and spots of four different patients are shown. [file Image_3.tiff]
